# Melioidosis in lower provincial Cambodia: A case series from a prospective study of sepsis in Takeo Province

**DOI:** 10.1371/journal.pntd.0005923

**Published:** 2017-09-13

**Authors:** Kevin L. Schully, Catherine M. Berjohn, Angela M. Prouty, Amitha Fitkariwala, Tin Som, Darith Sieng, Michael J. Gregory, Andrew Vaughn, Sim Kheng, Vantha Te, Christopher A. Duplessis, James V. Lawler, Danielle V. Clark

**Affiliations:** 1 Naval Medical Research Center, Biological Defense Research Directorate, Ft. Detrick, Maryland, United States of America; 2 Naval Medical Research Unit-2, Phnom Penh, Cambodia; 3 Cambodian Communicable Disease Control, Ministry of Health, Phnom Penh, Cambodia; 4 Takeo Provincial Referral Hospital, Takeo, Cambodia; University of Liverpool, UNITED KINGDOM

## Abstract

Melioidosis is a severe infectious disease caused by the gram-negative soil bacterium *Burkholderia pseudomallei*. Melioidosis is well known to be a major cause of morbidity and mortality in Southeast Asia, particularly in Thailand. However, melioidosis remains underreported in surrounding areas such as Cambodia. We report a case series of melioidosis in seven patients from Takeo Province, Cambodia. The patients, aged 24–65 years, were enrolled from May 2014 to May 2015 during a one year prospective study of sepsis at Takeo Provincial Hospital. They presented with fever, rigors, dyspnea, fatigue, diaphoresis, productive cough, and skin abscesses. Six of the seven patients were also hyponatremic. *B*. *pseudomallei* was cultured from the blood of six patients and the sputum of one patient. In this manuscript, we provide a detailed description of the clinical presentation, case management and laboratory confirmation of *B*. *pseudomallei*, as well as discuss the difficulties of identifying and treating melioidosis in low resource settings.

## Author summary

In spite of the fact that Cambodia shares borders with well-known hyper-endemic areas for *Burkholderia pseudomallei* where thousands of melioidosis cases occur each year, only a handful of melioidosis cases have been described in Cambodia. This is due, in part, to the lack of appropriate diagnostic resources there and the fact that physicians are not familiar with the appropriate diagnostic and treatment algorithms. As a result, the true burden of melioidosis in Cambodia is drastically underestimated and confirmed cases are rarely treated properly. During a one year study of sepsis in Takeo Provincial Hospital in Takeo Province Cambodia, we enrolled 139 patients which included seven culture-confirmed cases of melioidosis. As part of our study, laboratory workups including blood gasses, blood chemistries and hematology are routinely performed as well as imaging and a detailed record of medications administered during treatment. As a result, our report describes one of the more well-characterized cohorts of adult *B*. *pseudomallei* infections from Cambodia, originating in a region that had relatively few documented cases previously. Additionally, our case series touches upon a number of challenges faced by resource-limited health systems addressing melioidosis in areas of high endemicity.

## Introduction

The gram-negative soil bacterium *Burkholderia pseudomallei* is a facultative intracellular pathogen and the causative agent of melioidosis. The organism is highly endemic in tropical regions of Northern Australia and Southeast Asia, the most commonly recognized regional epidemiological hot spots [1], and recent *in silico* analysis suggests *B*. *pseudomallei* has a much broader distribution. Geographic information system modeling predicts the global burden of melioidosis, once thought to be a few thousand cases per year, to be 165,000 infected persons resulting in approximately 89,000 deaths annually [2]. *B*. *pseudomallei* is responsible for 20% of community-acquired blood stream infections in highly endemic regions [3], lagging only HIV and tuberculosis as the most common infectious cause of mortality in northeast Thailand [4]. Melioidosis is primarily contracted through contact with contaminated water and soil, [5] resulting in percutaneous inoculation, although ingestion [6–8] and inhalation [9, 10] also cause infection. Common risk factors for melioidosis in adults include occupational exposure to water and soil, diabetes mellitus, alcohol abuse, and other immunocompromising conditions.

Melioidosis often presents as pneumonia, but it can also cause blood stream infections or abscesses in almost any tissue compartment [1, 11]. Diagnosis of melioidosis is confounded by the protean manifestations of the disease ranging from acute, subacute and chronic infections which lack clear pathognomonic features. Acute melioidosis associated with primary infection is thought to represent over 85% of clinical cases [11] and is typically the most aggressive form. Symptoms can develop as early as one day following initial exposure, although the median incubation period is 9 days [12]. In endemic regions, acute melioidosis results in death in up to 40% of hospitalized patients, depending on available resources [4]. Chronic melioidosis affects approximately 11% of patients, resulting in symptoms lasting for months or even years [11, 13]. Recurrent disease is thought to affect approximately 1 in 16 melioidosis patients in endemic areas, with approximately 25% attributable to reinfection within 1 year, and the rest attributable to relapse [11, 14]. Outside of endemic areas, reactivation of latent *B*. *pseudomallei* is thought to be rare, but has been documented decades after the initial exposure [15–18].

Melioidosis in Southeast Asia was first identified in Burma in 1912 [19–21], followed by Malaysia and Singapore (1913), Vietnam (1925), Thailand (1955) [22], and Laos in 2001 [23]. The first endemic human case in Cambodia was not reported until 2008 [24]; although animal and exported human cases predate that first endemic case report [25–27]. Nevertheless, the true incidence in Cambodia is likely underrepresented in the scientific literature [24, 28]. Cambodia shares borders with known hyper-endemic regions in Thailand, where over 2,000 cases are estimated to occur per year [29], and 80% of the population demonstrate serologic evidence of exposure to *B*. *pseudomallei* by the age of four [30]. While in Cambodia, a single serosurvey in the Siem Reap Province detected antibodies in 16% of children [31] and recent reports have described only several hundred cases of melioidosis in 14 Cambodian provinces since 2007 [28, 32–37]. Although the true burden of *B*. *pseudomallei* remains uncertain, it is clearly a significant cause of sepsis and community-acquired pneumonia in Cambodia.

The Austere environments Consortium for Enhanced Sepsis Outcomes (ACESO) began an observational study of sepsis in Takeo Province, Cambodia in 2014. ACESO is comprised of university, non-profit, and US Department of Defense partners, headquartered at the Naval Medical Research Center, Frederick, Maryland (NMRC-F) and is focused on reducing fatality from sepsis in resource-limited settings. ACESO’s prospective cohort study of sepsis conducted at Takeo Provincial Hospital in Cambodia contributes to the discovery of host-biomarker profiles intended, in time, to direct clinical management interventions. Over the course of the first enrollment year of the study, seven culture-positive melioidosis cases were identified among 139 participating patients. Here, we present these initial seven melioidosis patients arising from four provinces in Cambodia and briefly review diagnosis and treatment of melioidosis in Cambodia.

## Methods

### Study site

**Fig 1:**
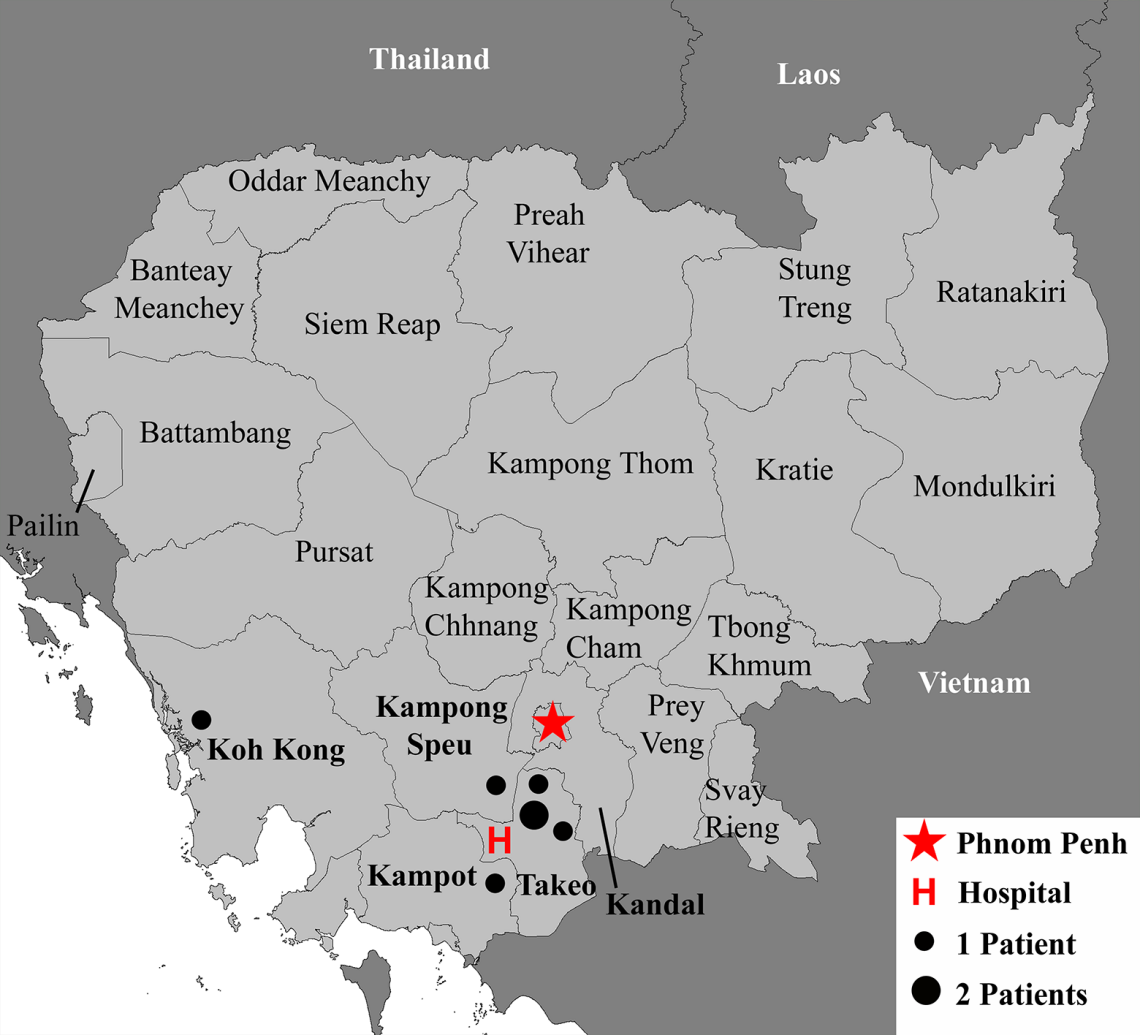
Study site. We used DIVA-GIS (http://diva-gis.org/) to create a map of Cambodia and surrounding areas. The red H indicates Takeo Provincial Hospital, the study site for this work. Dots indicate the location of the home village of seven melioidosis patients. Map is reflective of Cambodian Provincial borders during the time of patient enrollment.

All study participants were enrolled at Takeo Provincial Hospital, located in the Tram Kak District of Takeo Province (Fig 1, “H”). The hospital is the major regional government referral hospital, possessing approximately 250 beds and admitting roughly 800 patients per month. Takeo Province is located in the southwest of Cambodia (Fig 1), with a population of approximately 900,000 people [38]. The climate is tropical: warm and humid year-round, with a average annual temperatures of 29–36°C [39]. The wet season ranges from May through October and produces an average annual rainfall of 1250 mm [40]. The main agricultural product in Takeo and the surrounding areas is rain-fed lowland rice. Although the majority of the patients receiving care at Takeo Provincial Hospital are residents of Takeo Province, hospital administration estimates that approximately one-quarter come from neighboring provinces and Phnom Penh. In 2008, this hospital diagnosed the first endemic case of human melioidosis in Cambodia [24].

### Prospective enrollment

The ACESO prospective observational study of sepsis in Cambodia was approved by the Naval Medical Research Center Institutional Review Board in compliance with all applicable Federal regulations governing the protection of human subjects. Admission and treatment decisions were exclusively at the discretion of the hospital’s attending physicians. All patients, or their legally authorized representatives, provided written informed consent. The study considered for enrollment adult patients identified on admission with systemic inflammatory response syndrome (SIRS, defined as at least two of the following: temperature ≤ 35.5 or ≥ 37.5 C, heart rate ≥ 100/min, respiratory rate ≥ 20/min) attributed to infectious etiology. Full inclusion and exclusion criteria are included in S1 Table. Following informed consent, a detailed patient history was recorded and blood was drawn (10 ml from a single site) for culture and a variety of clinical analyses. Lithium heparinized whole venous blood was analyzed for lactate and electrolytes by the epoc (Alere, Waltham MA) point of care blood analysis system, for chemistries by the Piccolo Xpress Chemistry Analyzer (Abbott, Princeton, NJ), and for complete blood counts using the QBC Autoread Plus Dry Hematology System (Drucker Diagnostics, Port Matilda, PA).

### Bacteriology

The microbiology laboratory at Takeo Provincial Hospital is equipped with basic microbiology equipment including a standard incubator, biological safety cabinet, autoclave, microscope, refrigerators and freezers. Blood specimens, 10 ml drawn from a single site at the time of study enrollment, were cultured in blood culture bottles supplied by the Diagnostic Microbiology Development Program (DMDP) [41] (dmdp.org). Positive blood cultures with gram-negative organisms were sub-cultured on Ashdown’s medium, a selective and differential growth medium for *B*. *pseudomallei* [42, 43]. Sputum samples were plated directly onto Ashdown’s agar. Of note, the sputum culture capability was unavailable for the first seven months of the study. Isolates were confirmed through standard biochemical means (API 20NE system, bioMérieux) [44]. All seven isolates identified as *B*. *pseudomallei*; six as 1056577 and one as 1056574. Finally, molecular confirmation was obtained through real time PCR using primers specific for the Type III Secretion System of *B*. *pseudomallei* [45]. Manual antibiotic sensitivity testing was conducted employing the Kirby-Bauer disk diffusion method with breakpoints determined by the Diagnostic Microbiology Development Program based on the 2013 CLSI guidelines.

## Results

From May 30, 2014 through May 26, 2015, 139 patients were enrolled at Takeo Provincial Hospital. The median age was 49 years (range: 18–83), and males constituted 74.8%. From these patients, seven were culture-positive (six from blood, one from sputum only) for *B*. *pseudomallei*. All melioidosis patients were males between the ages of 24 and 65 years (median 47 years). All patients presented with fever and rigors. Dyspnea, fatigue, diaphoresis, productive cough, and skin abscesses were commonly co-occurring symptoms (S1 Table). Six of the seven patients were hyponatremic (Table 1) and five of the seven patients possessed typical risk factors such as diabetes (n = 3) and rice farming (n = 3). Five patients were enrolled during the rainy season, while two were enrolled during the dry season. Four fatalities occurred, and the median time to death from the onset of fever was 15.5 days (range 6–49). The clinical isolates exhibited expected biochemical profiles and antibiotic susceptibilities to ceftazidime, imipenem and amoxicillin/clavulanic acid (Table 2). One of our isolates was determined to be resistant to Trimethoprim/sulfamethoxazole.

**Table 1:** Lab results (A) and vital signs (B) collected at enrollment.

A	*1*	2***	*3*	4***	*5*	*6*	7*
***WBC(cells/μl)*** ***(3.5–10×10^3^)***	**11.7×10**^**3**^	**8.30×10**^**3**^	**29.2×10**^**3**^	**9.80×10**^**03**^	**11.3×10**^**3**^	**19.9×10**^**3**^	**18.5×10**^**3**^
***Granulocyte Count(cells/μl)*** ***(1.2–8×10^3^)***	**10.4×10**^**3**^	**6.40×10**^**3**^	**26.0×10**^**3**^	**8.0×10**^**3**^	**9.8×10**^**3**^	**18.0×10**^**3**^	**15.6×10**^**3**^
***Granulocyte %*** ***(35–80)***	**88**	**77**	**89**	**82**	**87**	**90**	**84**
***Lymphocyte/Monocyte Count (cells/μl)*** ***(0.5–5×10^3^)***	**1.3×10**^**3**^	**19.0×10**^**3**^	**3.2×10**^**3**^	**1.8×10**^**3**^	**1.5×10**^**3**^	**1.9×10**^**3**^	**2.9×10**^**03**^
***Lymphocyte/Monocyte %*** ***(15–50)***	**12**	**23**	**11**	**18**	**13**	**10**	**16**
***Hgb (g/dL)*** ***(11.5–16.5)***	**11.0**	**12.9**	**13.4**	**8.1**	**11.6**	**11.9**	**11.6**
***Hct (%)*** ***(38–51)***	**41**	**40**	**40**	**31**	**34**	**28**	**37**
***Platelet (per μl)*** ***(1–4×10^5^)***	**2.16×10**^**5**^	**2.01×10**^**5**^	**2.86×10**^**5**^	**1.76×10**^**5**^	**1.99×10**^**5**^	**1.82×10**^**5**^	**2.42×10**^**5**^
***Na+ (mmol/L)*** ***(128–145)***	**104**	**135**	**127**	**129**	**133**	**124**	**123**
***K+ (mmol/L)*** ***(3.6–5.1)***	**4.5**	**4.3**	**3.3**	**2.6**	**3.3**	**5.8**	**5.1**
***iCa++ (mmol/L)*** ***(1.15–1.33)***	**0.87**	**1.11**	**0.93**	**1.13**	**1.16**	**0.91**	**1.12**
***BUN (mg/dL)*** ***(7–22)***	**14**	**13**	**10**	**13**	**32**	**144**	**17**
***Glu (mg/dL)*** ***(72–100)***	**112**	**361**	**133**	**188**	**270**	**91**	**369**
***Cr (mg/dL)*** ***(0.6–1.2)***	**1.6**	**0.6**	**1.3**	**0.7**	**1.1**	**13.3**	**ICT**
***Alb (g/dL)*** ***(3.3–5.5)***	**2.4**	**2.5**	**2.9**	**2.0**	**1.8**	**2.4**	**2.5**
***ALT (u/L)*** ***(10–47)***	**153**	**96**	**9**	**34**	**32**	**88**	**150**
***AST (u/uL)*** ***(11–38)***	**313**	**56**	**22**	**45**	**64**	**52**	**169**
***Lac (mmol/L)*** ***(0.55–1.38)***	**4.31**	**2.58**	**3.66**	**6.47**	**2.65**	**1.81**	**4.88**
**B**							
***Temperature (°C)*** ***(36.1–37.2)***	**38.5**	**39**	**39**	**38.5**	**37**	**39**	**38.5**
***Heart Rate(beats per min)*** ***(60–100)***	**112**	**116**	**128**	**138**	**100**	**114**	**92**
***Respiratory Rate(breaths per min)*** ***(12–20)***	**28**	**34**	**28**	**26**	**22**	**30**	**20**
***Blood Pressure (mmHg)***	**140/90**	**100/70**	**100/70**	**120/80**	**80/50**	**190/100**	**140/90**

From QBC Autoread Plus Dry Hematology System: All cell counts including Granulocyte, Lymphocyte/Monocyte, WBC, White Blood Count; Hgb, Hemoglobin. From epoc: Lac, Lactate; Glu, Glucose; Hct, Hematocrit; Ca++, Total Calcium. From Piccolo Xpress Chemistry Analyzer: BUN, Blood Urea Nitrogen; Cr, Creatinine; ALB, Albumin; ALT, Alanine Aminotransferase; AST, Aspartate aminotransferase. ICT indicates interference from icterus. Expected normal values, as provided by the monitoring equipment, are given in parentheses below each parameter. Readings were collected at admission unless otherwise indicated (*+1 day, ***+3days).

**Table 2:** Clinical isolate characteristics.

	*1*	2***	*3*	4***	*5*	*6*	7*
***Source*** ***Time to culture positive (days)***	**Blood**	**Blood**	**Blood**	**Blood**	**Blood**	**Blood**	**Sputum**
	**2**	**5**	**1**	**1**	**5**	**4**	**7**^**#**^
***Ceftazidime(30)***	**S(22)**	**S(22)**	**S(24)**	**S(21)**	**S(23)**	**S(26)**	**ND**
***Imipenem(10)***	**S(31)**	**S(30)**	**S(32)**	**S(39)**	**S(32)**	**S(38)**	**ND**
***Amox-Clav (20/10)***	**S(22)**	**S(21)**	**S(21)**	**S(23)**	**S(19)**	**S(25)**	**ND**
***TMP/SMX(1.25/23.75)***	**S(>16)**	**S(>16)**	**S(>16)**	**S(>16)**	**S(>16)**	**R(6)**	**ND**

All clinical isolates grew on Ashdown’s medium and were gram-negative, bi-polar staining bacilli. Each isolate was oxidase positive, citrate positive and motile. Each isolate produced alkaline reactions on TSI and was violet on LIA in addition to being negative for Indole, H_2_S and gas production. For AST testing, the concentration (μg) of the antibiotic disk is in parentheses next to the antibiotic name. The zone of inhibition (mm) for each antibiotic is indicated in parentheses next to its results: S, Sensitive; R, Resistant. # denotes sputum culture was plated directly onto Ashdown’s Agar. Cultures were collected at admission unless otherwise indicated (*+1 day, ***+3days). Remaining Time to culture positive results indicate blood culture bottle positive.

### Case series

Patient 1 was a 65-year-old male office worker from Kampot Province (Fig 1). He was transferred in June to Takeo Provincial Hospital with pneumonia and a large abscess of the left arm (S1 Table). He had spent two days in his district hospital, where he presented with six days’ duration of cough and received gentamicin and amoxicillin. Upon enrollment at Takeo, he was febrile (39.5°C), tachycardic (112 bpm) and tachypneic (28 breaths per minute) with elevated blood pressure (140/90 mmHg). Laboratory tests performed on admission revealed a mild leukocytosis with left shift, anemia, marked hyponatremia (confirmed at the T6hr blood draw), mildly elevated creatinine, and elevated liver enzymes (Table 1A). He was treated with ceftriaxone (2g daily IV) and gentamicin (240mg IV). On the day of admission and against medical advice, the patient was taken home by family members, where he died the next day. Following his departure from the hospital, his blood culture became positive and an isolate subsequently grew on Ashdown’s agar. It was identified as *B*.*pseudomallei* through biochemical and molecular testing (Table 2).

**Fig 2:**
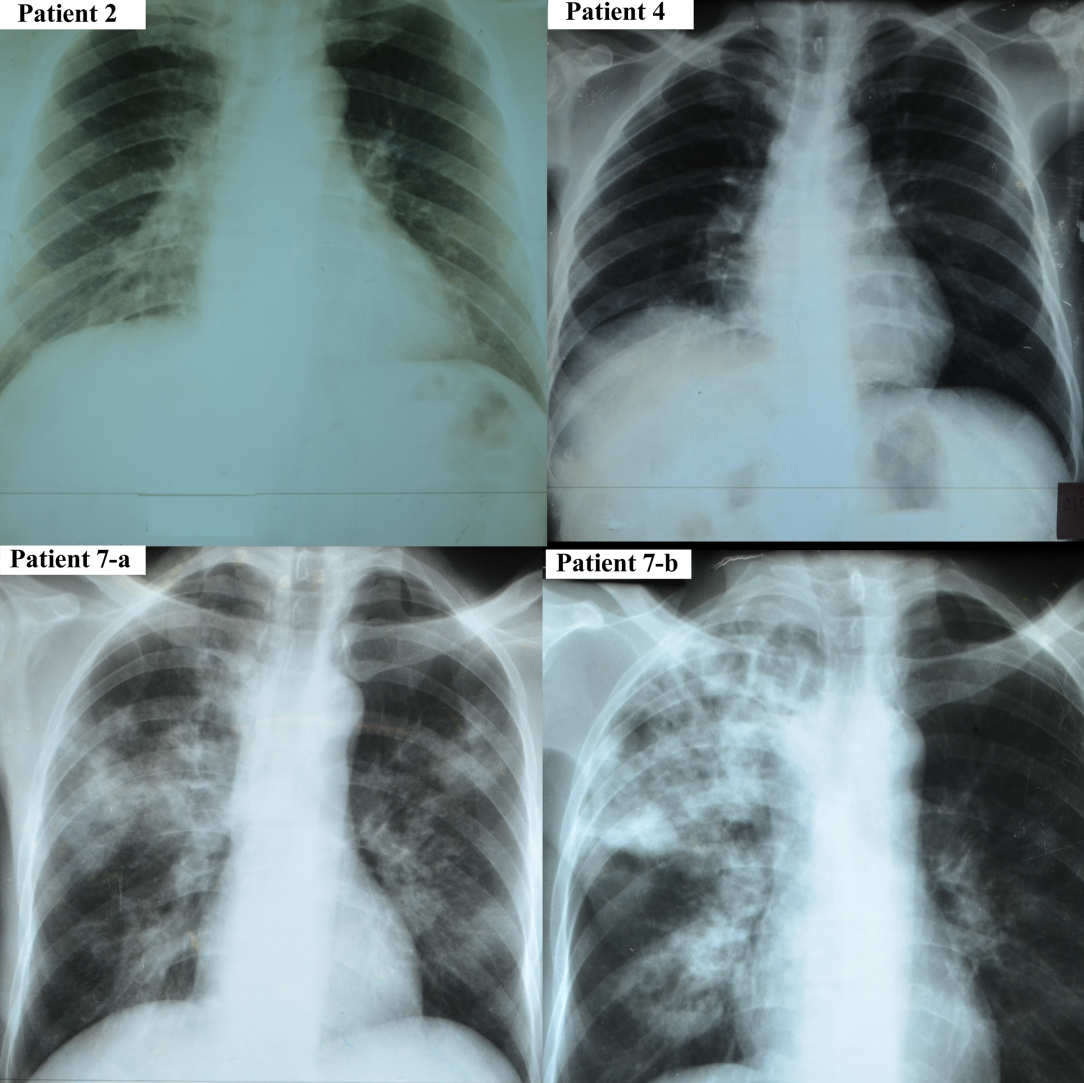
Chest radiography. Plain films taken on hospital day 1 (patient 2, 4, 7a) and hospital day 9 (patient 7-b).

Patient 2 was a 44-year-old male teacher from Takeo Province (Fig 1) who presented in June with (S1 Table) fever (39°C), tremors, dyspnea, palpitations, fatigue and diarrhea accompanied by (Table 1B) tachycardia (116 bpm) and tachypnea (34 breaths per minute). He did not carry a prior diagnosis of diabetes mellitus. However, a blood glucose on initial screening (hospital day two) was 361 mg/dL, which although confounded by the illness is highly suggestive of undiagnosed diabetes. Other than very mild elevation of liver-related enzymes, the rest of his laboratory values were unremarkable (Table 1A). He was initially treated with a three-day course of IV ampicillin (300mg), followed by one day of amoxicillin (3g PO) and ciprofloxacin (1g PO). The following day, he was switched to ceftriaxone (2g daily IV) and gentamicin (720mg IV), along with dihydroartemisinin-piperaquine for three days. An abdominal ultrasound was normal, while a chest x-ray revealed right middle lobe pneumonia (Fig 2). Seven days after admission, he was placed on intravenous ceftazidime (4 g IV) under the suspicion of melioidosis and transferred to Phnom Penh for further treatment. There, a chest x-ray revealed right upper lobe opacity and antimicrobial therapy consisted of ceftazidime (2g q12h IV), doxycycline (200 mg daily PO), and trimethoprim/sulfamethoxazole (3× 400/80 mg q8h PO). This regimen was maintained for a period of 10 days until the patient requested to return home. The patient was discharged with a course of doxycycline (200 mg daily PO), and trimethoprim/sulfamethoxazole (3× 400/80 mg q8h PO) for an unspecified amount of time. Three days after his transfer to Phnom Penh, the patient’s blood culture drawn at Takeo was positive. Subsequent isolation of the pathogen on Ashdown’s medium, coupled with molecular and biochemical characterization (Table 2), identified *B*. *pseudomallei*. At his 28 day follow-up, the patient reported improvement and has since made a full recovery from his infection.

Patient 3 was a 56-year-old male rice farmer from Takeo Province (Fig 1). He was admitted to hospital in June with fever (39°C), dyspnea, anorexia, malaise, abdominal pain, and diarrhea (S1 Table). Upon admission, he was tachycardic (128 bpm) and tachypneic (28 breaths per minute). Blood tests showed marked leukopenia with granulocyte predominance, hyponatremia, and hypokalemia (Table 1A). The patient was treated initially with ampicillin (1g IV q8h) and ceftriaxone (3g daily IV), followed by gentamicin (240 mg IV×1 dose). The patient died 25 hours after enrollment. The patient’s blood culture grew gram-negative bacilli 24 hours later, which were isolated on Ashdown’s medium. Biochemical and PCR identification of the isolate confirmed *B*. *pseudomallei* (Table 2).

Patient 4 was a 46-year-old rice farmer from Kampong Speu Province (Fig 1) who was admitted in July complaining of fever (38.5°C), rigors, dizziness, dyspnea, sweating, palpitations, and abdominal pain (S1 Table) as well as myalgias and arthralgias in his right shoulder for 28 days. He was enrolled in our study on hospital day two and was found to be tachycardic (138 bpm), and tachypenic (26 breaths per minute) with normal blood pressure (120/80 mmHg). Chest radiography (Fig 2) was consistent with right middle lobe pneumonia. Laboratory analysis revealed anemia (Hb 8.1 g/dL), hyponatremia, marked hypokalemia (K+ 2.6 mmol/L), and elevated serum glucose of 188 mg/dL (Table 1A). He was initially treated with ampicillin (1g IV q8h) for one day and gentamicin (80 mg IV q24h) for three days. An abdominal ultrasound revealed hepatosplenomegaly. Blood culture was positive for gram-negative rods. Intravenous ceftazidime (2g q8h IV) was begun on hospital day two for a period of 16 days, accompanied by trimethoprim/sulfamethoxazole (400/80 mg q12h PO). That antimicrobial regimen was followed by oral amoxicillin/clavulanate (2×500/125 q8h PO) for an unspecified duration. The blood culture isolate grew on Ashdown’s medium and was confirmed as *B*. *pseudomallei* by biochemical (Table 2) and PCR analysis. At his 28-day follow-up, the patient continued to suffer from fatigue and diaphoresis, as well as lingering joint pain, both of which persisted at least through his six-month visit. However, one year after treatment, the patient reported feeling fully recovered.

Patient 5 was a 48-year-old civil servant from Takeo Province (Fig 1). Although afebrile at presentation, he was admitted in August with complaints of intermittent fevers, anorexia, profuse diaphoresis, dyspnea, right upper quadrant pain, polydipsia, polyuria, and malaise (S1 Table). He had no known prior medical history, but screening at presentation revealed a random blood glucose of 270 mg/dL, potentially indicating diabetes (Table 1A). He was hypotensive at presentation (80/50 mmHg; Table 1B). His laboratory findings were only mildly abnormal (Table 1A). An ultrasound was consistent with multiple liver abscesses. The patient was treated initially with ceftriaxone (2g daily IV) for a period of five days, and on his sixth day of admission, the patient was transferred to Phnom Penh for further treatment. There, metronidazole (500 mg q8h IV), moxifloxacin (400 mg daily IV), and cefixime (2g IV × 1 dose) were administered. The following day, the cefixime was replaced with piperacillin/tazobactam (2 g/250 mg q8h IV). This antibiotic regimen was maintained for the duration of his admission, and he ultimately died on hospital day 11. Blood cultures from his initial admission in Takeo grew gram-negative bacilli on hospital day five. Isolation on Ashdown’s medium and subsequent biochemical and molecular characterization (Table 2) confirmed *B*. *pseudomallei*.

Patient 6 was a previously healthy 24-year-old male factory worker from Koh Kong Province (Fig 1) who was admitted to Takeo in December with complaints of fever (39°C), rigors, dizziness, headache, dyspnea, palpitations, cough, arthralgias, myalgias, fatigue, anorexia, abdominal pain, and edema (S1 Table). Additionally, he was tachycardic (114 bpm), tachypenic (30 breaths per minute) and hypertensive (190/100 mmHg) (Table 1B). Admission laboratory testing (Table 1A) revealed leukocytosis, anemia, hyponatremia, hyperkalemia, and markedly elevated BUN and Cr indicative of acute renal injury. At Takeo, blood cultures were drawn, and ceftriaxone (2g daily IV) was administered. An abdominal ultrasound revealed ascites. He was transferred that same day to a hospital in Phnom Penh, where he received a single dose of amoxicillin/clavulanic acid followed by 14 days of meropenem (1g q8h IV). In addition to the meropenem, he received moxifloxacin (400 mg IV q24h) for five days, followed by ciprofloxacin (200mg q12h IV) for the remaining nine days. On hospital day 20, the patient was discharged at his own request. A follow up call from Takeo Hospital approximately three weeks later revealed that the patient had died at his home. Blood cultures drawn at admission were positive after four days, and ultimately confirmed as *B*. *pseudomallei*.

Patient 7 was a 46-year-old previously healthy male rice farmer from Takeo Province (Fig 1) who was admitted in January with fever, rigors, diaphoresis, fatigue, dyspnea, palpitations, productive cough, anorexia, myalgias, and abdominal pain (S1 Table). Admission vital signs were temperature 38.5°C, heart rate 92 bpm, respiration 20 breaths per minute and blood pressure 140/90 mmHg (Table 1B). Auscultation revealed rales in both lungs, and chest radiography (Fig 2) confirmed lobar pneumonia. Laboratory examination (Table 2) revealed leukocytosis with neutrophil predominance, mild anemia, marked hyponatremia, hyperglycemia (random; 369 mg/dL) and elevated liver enzymes (4-fold). Sputum was collected approximately 20 hours after enrollment and plated directly on Ashdown’s agar, from which *B*. *pseudomallei* was isolated. Blood culture remained negative. The patient was initially treated with ceftriaxone (2g daily IV), with the addition of gentamicin (240 mg IV × 1 dose) the next day, followed by three days of amoxicillin/clavulanate (2× 500/125 mg q8h PO) and trimethoprim/sulfamethoxazole (2× 400/80 mg q8h PO) in place of gentamicin. On hospital day four, antibiotics were changed to a two-week course of intravenous ceftazidime (2g q8h IV), followed by a two-week course of oral amoxicillin/clavulanate and trimethoprim/sulfamethoxazole (2× 400/80 mg q8h PO). At his 28-day follow-up, the patient reported persistence of a productive cough but reported resolution of his other symptoms. At six months, he reported mild abdominal pain, which had resolved by the time of his 12-month visit.

## Discussion

Melioidosis is an important cause of sepsis in highly-endemic regions that is particularly critical to recognize and treat early because empiric treatment regimens fail to appropriately cover this organism. Melioidosis also likely represents an under-recognized problem in tropical latitudes across the globe [2]. Unfortunately, a majority of countries in high-risk regions are categorized as low- and middle-income and lack the health system resources required to evaluate and treat melioidosis appropriately. Improved awareness and understanding of this disease is essential to characterize the true global public health. Additionally, a higher index of suspicion will lead to earlier diagnoses and likely better outcomes, especially if sufficient quantities of appropriate antibiotic therapies are available.

This report describes one of the more well-characterized cohorts of adult *B*. *pseudomallei* infections from Cambodia, originating in a region that had relatively few documented cases previously. Our cases possess many features that are consistent with classical representations of melioidosis. All patients were male, corresponding to a predominance or males seen in previous studies, perhaps attributable to an increased prevalence of occupational and other behavioral risk factors [5]. Although isolated glucose elevations do not confirm the diagnosis of diabetes in severely ill patients, three patients presented with random glucose levels highly suggestive of diabetes mellitus, a comorbidity known to increase the risk of melioidosis [5]. With this in mind, three patients had overt diabetes, three patients had the known occupational risk of rice farming, and one patient possessed both of these risk factors. Clinically, all seven patients presented with fever and respiratory symptoms (shortness of breath and/or cough), and at least three were clearly diagnosed with pneumonia. Our case fatality proportion of 57% (4/7) is roughly consistent with previous case series in low-resource settings. Laboratory findings were also generally consistent with severe bacterial infection, although leukocytosis was not prominent, with normal or mildly elevated WBC counts occuring in 57% (4/7) of patients. Mild-to-moderate anemia and mild elevations of liver-associated enzymes in our patients were consistent with sepsis syndrome, as were the low serum ionized calcium levels and high frequency of elevated peripheral venous lactate levels (86%; 6/7) [46].

The presence of hyponatremia in all but one of our patients was one finding of particular interest. Basic serum chemistries are available in many hospitals in low- and middle-income regions and are thus readily available to assist in clinical diagnosis. Of our patients, 71% (5/7) had a Na < 130 mmol/L. Although hyponatremia is a frequent feature of sepsis and critical illness in general, and is particularly associated with pulmonary disease through the mechanism of the syndrome of inappropriate anti-diuretic hormone secretion (SIADH) and others, the prevalence and degree of hyponatremia in our patients who had symptoms of pneumonia for < 1 week is somewhat disproportionate to the expected and may represent a distinguishing feature [47]. Hyponatremia has been offered as a similar clinical clue in other infectious diseases such as Legionnaire’s disease [48]. It is impossible to draw conclusions from this small case series, however, more research is warranted to discern the true association of hyponatremia and melioidosis, the underlying mechanism, and the potential utility as a diagnostic indicator for early recognition of melioidosis in resource-limited settings.

The diagnosis of melioidosis can be difficult and requires astute clinical and microbiologic evaluation. Because the clinical presentation of melioidosis is protean, microbiologic evidence of infection remains the gold standard [22]. In addition to blood, bodily fluid cultures including urine [49] and sputum [24, 50], as well as throat [51] and wound [52] swabs should be obtained routinely on suspected patients. Cultures from non-sterile sites should be grown in/on selective and differential media (i.e., Ashdown’s medium [42]) to differentiate *B*. *pseudomallei* from normal flora [52]. Clinicians should alert laboratory staff to a suspicion of *B*. *pseudomallei* to pursue appropriate targeted microbiologic testing and adopt appropriate safeguards against this biohazard. Even with an appropriate index of suspicion and a reliable capability for isolating and identifying *B*. *pseudomallei*, microbiologic confirmation is often too slow. In our series, two of four fatalities occurred prior to growth and identification of the organism from blood cultures. This experience emphasizes the importance of more aggressive cultures from multiple sites with increased use of Gram stain for presumptive diagnosis, as well as modifications to empiric coverage when the index of suspicion is high. It also argues for continued research and development in alternative diagnostic assays for *B*. *pseudomallei* infection that possess better sensitivity and more rapid results.

Accurate diagnosis is critically important in melioidosis because appropriate antimicrobial treatment varies greatly from typical empiric regimens. *Burkholderia pseudomallei* is intrinsically resistant to an array of antimicrobial agents, which coupled to its intracellular nature makes melioidosis extremely difficult to treat [22, 53, 54]. Intravenous ceftazidime or a carbapenem (imipenem or meropenem) are the mainstays of initial intensive therapy for melioidosis, as there is convincing evidence of mortality reduction compared to more standard antimicrobial agents [22, 55]. In many highly-endemic areas, empiric antimicrobial therapy with ceftazidime or a carbapenem may be warranted for appropriate clinical syndromes. Unfortunately, many hospitals in these regions are not adequately resourced to adopt this approach. For example, at the time of this manuscript’s preparation, Takeo Provincial Referral Hospital’s annual allotment of formulary antimicrobials included enough ceftazidime to properly treat only 12–14 melioidosis patients. Additionally, a recent survey revealed that only 17% of Cambodian physicians have any experience treating melioidosis [56]. Although numbers are too small to draw significant statistical conclusions from our series, it is interesting to note that of the four patients who survived long enough to receive a course of ceftazidime or meropenem, three survived.

Although studies have reported resistance to first-line antimicrobial agents for melioidosis, no resistance to ceftazidime or meropenem was observed in our isolates. Resistance to trimethoprim/sulfamethoxazole was also evaluated and one isolate was found to be resistant (Table 2). However, using disc diffusion testing may overestimate the resistance rate of TMP/SMX *B*. *pseudomallei*; therefore Etest is recommended for TMP/SMX testing [57]. We recommend that antibiotic susceptibility testing become standard practice to monitor for emergence of resistance and to guide longer term outpatient oral maintenance therapy.

Our prospective observational study identified 7 culture-confirmed cases of melioidosis out of 139 patients presenting with a clinical diagnosis of sepsis in a regional referral hospital in Cambodia. Our finding confirms previous studies that identified *B*. *pseudomallei* as an important cause of pneumonia and sepsis in Cambodia. We suspect that there are undiagnosed cases of melioidosis in our cohort and that the 5% prevalence substantially underestimates the true prevalence in this cohort. This underestimation is due, in part, to the fact that we introduced Ashdown’s medium and sputum culture approximately seven months into the study and blood was the only specimen cultured for the first 80 patients enrolled. As we continue to work with the study site hospital to increase non-blood site cultures and institute additional diagnostic assays for *B*. *pseudomallei*, we expect our proportion of laboratory confirmed cases to increase significantly.

Our case series provides insight into a few of the numerous challenges faced by resource-limited health systems addressing melioidosis in areas of high endemicity. Limited epidemiological data and laboratory testing capacity in probable high-risk areas hamper early clinical suspicion and microbiologic confirmation of melioidosis. Inadequate formularies restrict availability of effective antimicrobials against *B*. *pseudomallei*. A lack of antibiotic stewardship programs and treatment guidelines can also result in unnecessary waste of these scarce antimicrobials. Limited central venous access and inadequate systems for outpatient antibiotic infusion can also contribute to frequent under-treatment of acute infection. Finally, systemic and economic barriers to long-term, close follow-up impedes medication adherence for the recommended 3–6 months of oral therapy. While these problems are daunting, concerted and coordinated efforts by researchers, clinicians, laboratorians, health system administrators, and public health officers can address them and make a profound impact in patient outcomes for melioidosis. Increased international advocacy, funding, and government action is necessary to combat this important tropical disease.

## Supporting information

S1 Table Patient-reported symptoms. Complaints of symptoms and duration (days) prior to hospitalization were collected during enrollment. Symptoms were collected at admission unless otherwise indicated (*+1 day, ***+3days).
(DOCX)

S2 Table Supplemental clinical data. ^a^Number of days from onset of fever until admission to Takeo Regional Referral Hospital; ^b^Number of days from admission to TRRH to death; ^c^Sequential Organ Failure Assessment (SOFA) Score calculated at enrollment into study.
(DOCX)
