# Supplementary material for: Melioidosis in lower provincial Cambodia: A case series from a prospective study of sepsis in Takeo Province
Source: PLoS Negl Trop Dis. 2017 Sep 13;11(9):e0005923. doi: 10.1371/journal.pntd.0005923 (PMC5612750; doi:10.1371/journal.pntd.0005923)
Supplement: S1 Table — Complaints of symptoms and duration (days) prior to hospitalization were collected during enrollment. Symptoms were collected at admission unless otherwise indicated (*+1 day, ***+3days). (DOCX) [file pntd.0005923.s001.docx]

|  | **1** | **2***** | **3** | **4***** | **5** | **6** | **7*** |
| --- | --- | --- | --- | --- | --- | --- | --- |
| **Fever** | **6** | **12** | **5** | **4** | **15** | **4** | **4** |
| **Rigors** | **6** | **12** | **5** | **4** | **7** | **4** | **4** |
| **Diaphoresis** | **6** | **12** |  | **4** | **7** |  | **4** |
| **Dizziness** | **6** |  | **3** | **4** |  | **4** |  |
| **Dyspnea** | **3** | **10** | **7** | **4** |  | **4** | **4** |
| **Palpitations** |  | **6** | **7** | **4** |  | **4** | **4** |
| **Productive Cough** | **6** |  | **7** |  |  | **4** | **4** |
| **Fatigue** | **3** | **12** | **2** | **4** |  | **4** | **4** |
| **Anorexia** |  | **12** | **2** |  |  | **4** | **4** |
| **Nausea** | **3** |  | **1** |  |  |  |  |
| **Diarrhea** |  | **5** | **1** |  |  |  |  |
| **Abdominal Pain** |  |  | **3** | **4** |  | **4** | **4** |
