# Supplementary material for: Melioidosis in lower provincial Cambodia: A case series from a prospective study of sepsis in Takeo Province
Source: PLoS Negl Trop Dis. 2017 Sep 13;11(9):e0005923. doi: 10.1371/journal.pntd.0005923 (PMC5612750; doi:10.1371/journal.pntd.0005923)
Supplement: S2 Table — aNumber of days from onset of fever until admission to Takeo Regional Referral Hospital; bNumber of days from admission to TRRH to death; cSequential Organ Failure Assessment (SOFA) Score calculated at enrollment into study. (DOCX) [file pntd.0005923.s002.docx]

| **Patient** | **Fever to admit**^a^ | **Admit to death**^b^ | **SOFA score**^c^ |
| --- | --- | --- | --- |
| **1** | 6 | 1 | 4 |
| **2** | 12 | N/A | 1 |
| **3** | 5 | 1 | 1 |
| **4** | 4 | N/A | 1 |
| **5** | 15 | 9 | 1 |
| **6** | 4 | 45 | 5 |
| **7** | 4 | N/A | 4 |
